# Comparative performance of the patient-generated subjective global assessment, European Society for Clinical Nutrition and Metabolism criteria, and Global Leadership Initiative on Malnutrition criteria in patients with colorectal cancer: a multicenter study utilizing Bayesian inference

**DOI:** 10.3389/fnut.2025.1671154

**Published:** 2026-02-16

**Authors:** Ze-cheng Zhang, Wei-dong Jin, Hong-jiang Ma, Yi Zhang, Yunlong Li, Jun Zhu, Yi-huan Qiao, Yong-tao Du, Yu Jiang, Jia-wei Song, Jia-lin Wang, Shuai Liu, Ya-jie Guo, Bo-yu Kang, Qi Wang, Shi-hao Qin, Chun-hua Song, Han-ping Shi, Ji-peng Li

**Affiliations:** 1 Department of Digestive Surgery, Xijing Hospital of Digestive Diseases, Air Force Medical University, Xi’an, China; 2 Department of Experimental Surgery, Xijing Hospital, Air Force Medical University, Xi’an, China; 3 Department of General Surgery, General Hospital of Central Theater Command, Wuhan, China; 4 Department of Epidemiology, College of Public Health, Zhengzhou University, Zhengzhou, China; 5 Department of Gastrointestinal Surgery/Department of Clinical Nutrition, Beijing Shijitan Hospital, Capital Medical University, Beijing, China

**Keywords:** colorectal cancer, Patient-Generated Subjective Global Assessment, European Society for Clinical Nutrition and Metabolism, Global Leadership Initiative on Malnutrition, malnutrition, diagnosis, Bayesian

## Abstract

**Background:**

Malnutrition is a common complication among patients with colorectal cancer and has a significant impact on prognosis. However, the lack of a diagnostic gold standard for malnutrition complicates clinical nutritional intervention. This study aimed to compare the diagnostic value of the Patient-Generated Subjective Global Assessment (PG-SGA), European Society for Clinical Nutrition and Metabolism (ESPEN), and Global Leadership Initiative on Malnutrition (GLIM) criteria in identifying malnutrition in patients with colorectal cancer and to assess their ability to predict survival outcomes.

**Methods:**

This study retrospectively reviewed data from 3,182 patients diagnosed with colorectal cancer in the Investigation on Nutrition Status and Clinical Outcome of Patients with Common Cancers in China database collected between July 2013 and March 2022. Using Bayesian principles, we calculated the sensitivity and specificity of the PG-SGA, ESPEN, and GLIM criteria for diagnosing malnutrition among patients with colorectal cancer. We also analyzed the relation between nutritional diagnosis and patient survival.

**Results:**

Our findings revealed that the PG-SGA has high sensitivity [0.80, 95% credible interval (CrI): 0.61–0.94] and specificity (0.99, 95% CrI, 0.99–1.00) for diagnosing malnutrition in patients with colorectal cancer. The ESPEN criteria showed high sensitivity (0.84, 95% CrI, 0.80–0.86), whereas the GLIM criteria exhibited high specificity (0.81, 95% CrI, 0.79–0.82). All three nutritional assessment methods were identified as independent risk factors for overall survival. Statistically significant differences in survival periods existed among the GLIM-defined nutritional status subgroups.

**Conclusion:**

The PG-SGA demonstrated superior sensitivity and specificity in diagnosing malnutrition among patients with colorectal cancer. By contrast, the GLIM criteria performed better in predicting survival outcomes. Malnutrition is a significant risk factor that influences the survival of patients with colorectal cancer.

## 1 Introduction

Malignant tumors are a critical health issue in contemporary populations. According to global health data, these diseases are among the leading causes of premature death and impose a substantial burden on public health systems worldwide (1). Malnutrition is a common complication associated with malignant tumors and represents one of the primary reasons for poor prognosis in patients with malignant tumors (2). Colorectal cancer (CRC) is one of the most common malignant tumors: it is the third most common cancer worldwide and ranks as the second most common cause of cancer-related deaths globally. In China, it is the third most common cancer and the fifth leading cause of cancer-related deaths (3). The Investigation on Nutrition Status and Clinical Outcome of Patients with Common Cancers in China (INSCOC) study showed that the severity of malnutrition in patients with CRC was one of the highest among all malignancies (4).

An accurate diagnosis of malnutrition is essential for proper nutritional intervention (5). Several nutritional assessment tools are available, but the Patient-Generated Subjective Global Assessment (PG-SGA) is currently the most widely used tool for assessing the nutritional status of patients (6, 7). However, its complexity poses challenges for routine clinical application (8). The European Society for Clinical Nutrition and Metabolism (ESPEN) proposed the ESPEN criteria for malnutrition in 2015 (9). This assessment method has been widely used in several studies and further evaluated in tumor populations (9–13). In 2018, the world’s four major nutritional societies jointly released the Global Leadership Initiative on Malnutrition (GLIM) criteria, which, to a certain extent, harmonized the diagnostic criteria for malnutrition (14). Since the release of the GLIM criteria, a substantial body of research has progressively validated its diagnostic value and prognostic significance (15–17).

Although various nutritional assessment methods have been published, there is still no recognized “gold standard” for nutritional assessment. The use of various nutritional assessment modalities remains highly controversial, particularly when applied to different cancer types.

Thus, this study aimed to compare the diagnostic value of the PG-SGA, ESPEN, and GLIM criteria for malnutrition in CRC patients and their ability to predict survival outcomes. This study aims to provide a theoretical basis for determining which nutritional assessment tools are most suitable as the “gold standard” for malnutrition diagnosis.

## 2 Materials and methods

### 2.1 Patient selection

The population included in this study was selected from the INSCOC project (trial registration number: ChiCTR1800020329), which collected data on 12,076 patients with CRC from multiple medical centers throughout China between July 2013 and March 2022. The registry of the study is Chinese Clinical Trial Registry (ChiCTR), and data of registration is from the website of ChiCTR.^1^ The registration date is November 10, 2011. The study design, technical route, inclusion criteria, and exclusion criteria of the INSCOC project have been published in previous studies (18, 19). This study complied with the Declaration of Helsinki and was approved by the ethics committees of each hospital.

### 2.2 Data collection

Data were collected from patient case report forms. The items included basic patient information, clinical examination, nutritional risk screening, nutritional assessment, the Quality of Life Scale for patients with cancer, nutritional support during hospitalization, indicators of recent clinical outcomes, self-assessments by investigators, and follow-up information. Laboratory tests were summarized using binary variables, and for most patients, these tests were within the normal range.

Basic patient information was obtained by one or two trained research professionals within 48 h of admission and included, among other things, the patient’s age, sex, personal medical history, family history of cancer, lifestyle habits, disease status, and Karnofsky Performance Status (KPS) score (20). The clinical tests included routine blood tests, blood biochemistry tests, anthropometric measurements, and compositional analyses. Nutritional risk screening was performed using the Nutritional Risk Screening 2002 tool (21). Nutritional assessment was conducted using the PG-SGA score, ESPEN criteria, and GLIM criteria (6, 9, 14). The Quality of Life Scale for patients with cancer was assessed using the Global Quality of Life by the European Organization for Research and Treatment of Cancer QLQ-C30 score (22). Recent clinical outcome metrics included the 30-d outcomes of the current admission, length of stay, hospital costs, and weight 30 d after admission. The follow-up records included the date of follow-up, visit status, survival status, cause of death, weight, body mass index (BMI), and KPS scores for up to 10 follow-up visits after the initial investigation, with a 1-year interval between each visit. The median follow-up time was 36.0 months. When patients require treatments such as surgery or preoperative chemoradiotherapy that necessitate hospitalization, hospitalization becomes necessary for the administration of these treatments.

### 2.3 PG-SGA

The PG-SGA comprises seven components: 1. weight loss; 2. food intake; 3. symptoms; 4. activity and physical function; 5. disease; 6.metabolic needs, and 7.physical examination. Typically, patient’s complete 1–4 items and medical staff score them, with the remaining items (5–7) completed and scored by medical staff. Nutritional status is categorized based on the PG-SGA score: 0–1 points indicates well-nourished, 2–3 points mildly malnourished, 4–8 points moderately malnourished, and ≥9 points severely malnourished. A score of ≥2 points suggests malnutrition and necessitates interventions such as nutritional support and monitoring (6). Healthcare professionals using the PG-SGA scoring system have received specialized training from the INSCOC project team.

### 2.4 ESPEN criteria

The ESPEN criteria refer to the Expert Consensus on Malnutrition Diagnosis published by ESPEN in 2015 (23). For patients with a positive nutritional screen, malnutrition was diagnosed when any of the following conditions were met: 1. BMI < 18.5 kg/m^2^. 2. Weight loss (unintentional) > 10% at any time or >5% within the last 3 months combined with BMI < 20 kg/m^2^ if <70 years of age or <22 kg/m^2^ if ≥70 years of age. 3. Weight loss (unintentional) > 10% at any time or >5% within the last 3 months combined with FFMI <15 and 17 kg/m^2^ for women and men, respectively. The measurement of FFMI is conducted using BIA.

### 2.5 GLIM criteria

We strictly followed the GLIM guidelines to conduct a thorough nutritional assessment (14, 24, 25). Patients who screened positive for nutritional risk proceeded to the second-step assessment, which encompassed phenotypic and etiological criteria. Phenotypic criteria included involuntary weight loss, low BMI, and reduced muscle mass. Etiologic criteria included reduced food intake or assimilation and inflammation or disease burden.

Unintentional weight loss was defined as >5% within the past 6 months or >10% beyond 6 months, based on nutritional interviews. For Asian populations, low BMI was defined as <18.5 kg/m^2^ for patients aged <70 years or <20 kg/m^2^ for those ≥70 years.

Reduced muscle mass was assessed using a pragmatic approach: we accepted either anthropometric measurements (calf and mid-arm circumference, as also included in the PG-SGA physical examination) or the Fat-Free Mass Index (FFMI), applying the same thresholds as in the ESPEN criteria (section 2.4). A patient was considered to have reduced muscle mass if they met the criteria by either method, whichever data were available. This approach ensured comprehensive assessment while accommodating clinical data availability.

Regarding the etiologic criterion of inflammation, in accordance with contemporary oncological understanding that characterizes cancer as a systemic disease with chronic inflammatory components (26), we considered the presence of the underlying malignant tumor as meeting this criterion for all patients in this cancer cohort.

Malnutrition was diagnosed when at least one phenotypic and one etiological criterion were met. Severity was graded primarily based on phenotypic criteria: moderate malnutrition was defined as 5–10% weight loss within 6 months or 10–20% over more than 6 months, and severe malnutrition as ≥10% within 6 months or ≥20% over more than 6 months.

### 2.6 Statistical analysis

The Shapiro–Wilk test was used to determine whether the variables conformed to a normal distribution. Data were presented as the median (interquartile range), mean (standard deviation), or number of cases (percentage). Categorical variables were compared using the chi-squared test, and Fisher’s exact test was used if the expected frequency in some cells was less than five. In this study, no cells had expected frequencies of <5. Missing data were filled using the mode imputation method. Since there is currently no universally accepted “gold standard” for diagnosing malnutrition, a comparative evaluation of the diagnostic value of various nutritional assessment tools was conducted using Bayesian principles (Supplementary material 1). This approach enabled the integration of prior knowledge with new evidence to update probability estimates for malnutrition diagnosis. The prior parameters were set based on previous studies. Kaplan–Meier curves and log-rank tests were used to display time-to-event data and compare survival outcomes between the groups. The median overall survival was calculated using Kaplan–Meier analysis. Using a multivariate Cox proportional risk model, hazard ratios (HRs) with 95% confidence intervals (CIs) were calculated to assess the correlation between malnutrition as diagnosed by different diagnostic methods and survival. Statistical significance was defined as *p* < 0.05 (two-sided). A sensitivity analysis was conducted to assess the potential impact of missing data imputation. We created a complete-case dataset by excluding subjects with any missing values in the baseline characteristics. The baseline profiles and the primary Cox regression survival analysis were then repeated and compared between this complete-case dataset (*n* = 2,881) and the primary imputed dataset (*n* = 3,182). All analyses were performed using SPSS (version 27; IBM Corporation, United States) and R.^2^

## 3 Results

### 3.1 Baseline characteristics of the population

**Figure 1:**
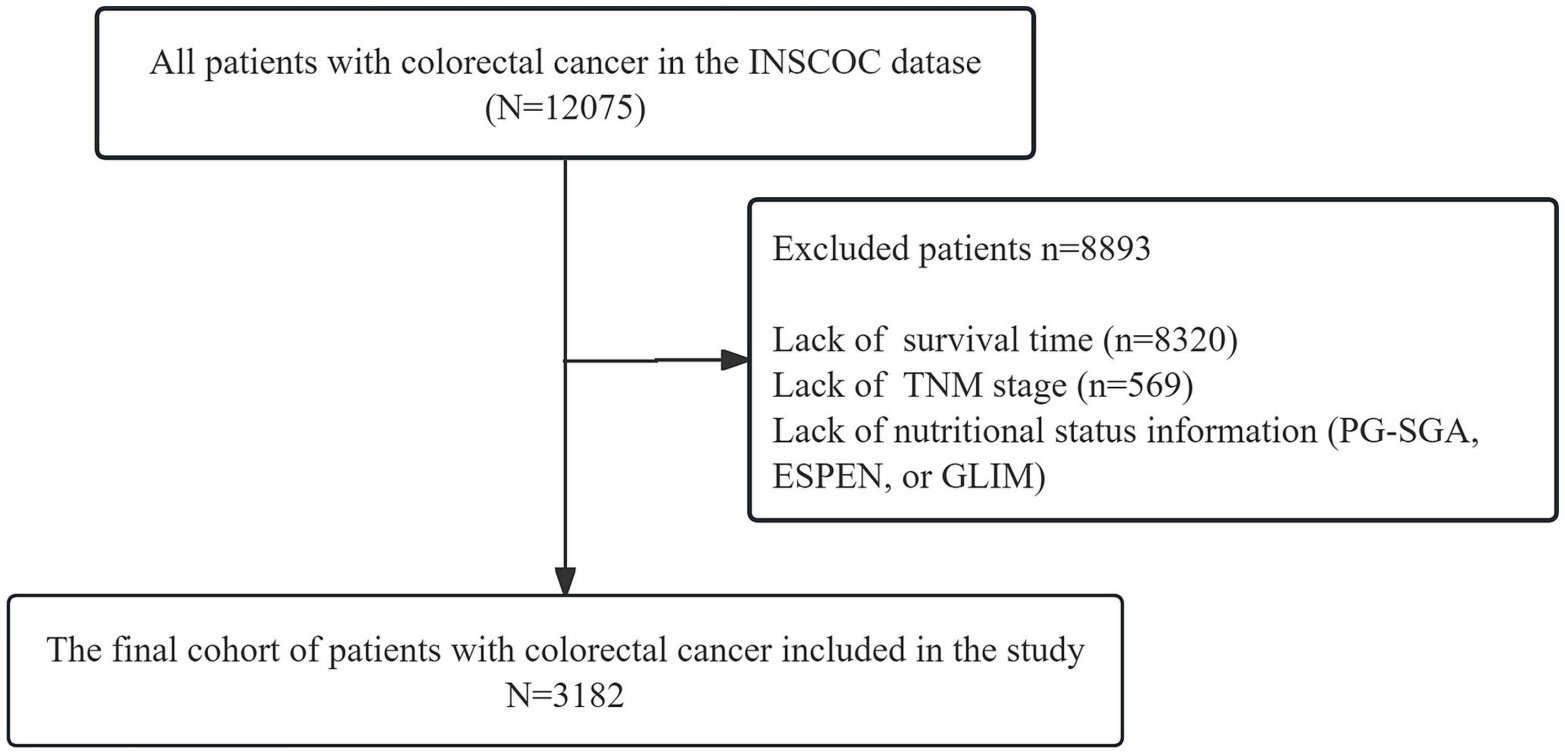
Flow chart of the patient inclusion process. INSCOC, investigation on nutrition status and clinical outcome of patients with common cancers in China.

The detailed patient selection process is illustrated in Figure 1. Briefly, from the initial INSCOC cohort, we excluded patients with missing overall survival data, missing TNM stage, or missing information for all three nutritional assessments (PG-SGA, ESPEN, and GLIM), as these were essential for the primary analysis. Consequently, the final number of patients enrolled in the study was 3,182. The baseline characteristics (Table 1) provide insights into the demographic and clinical profiles of the study population. Among the 3,182 participants, 58.5% were above the age of 65 years, and 59.7% were men. Most participants reported no history of smoking (62.1%) or alcohol consumption (82.6%), 21.2% had hypertension, and only 3.8% had coronary heart disease. A significant proportion of the population (9.6%) had diabetes mellitus. Additionally, the prevalence of malnutrition diagnosed using the PG-SGA and GLIM criteria was similar (19.1 and 19.9%, respectively), whereas the prevalence of malnutrition identified using the ESPEN criteria was notably high (53%).

**Table 1:** Patient demographics and baseline characteristics.

Characteristic	*N* = 3,182^1^
Age	
<65	1,322 (41.5%)
≥65	1,860 (58.5%)
Sex	
Male	1,901 (59.7%)
Female	1,281 (40.3%)
Diabetes	
No	2,877 (90.4%)
Yes	305 (9.8%)
Hypertension	
No	2,508 (78.8%)
Yes	674 (21.2%)
Coronary heart disease	
No	3,080 (96.2%)
Yes	122 (3.8%)
Smoke	
No	1,977 (62.1%)
Yes	1,205 (37.9%)
Alcohol	
No	2,827 (82.6%)
Yes	555 (17.4%)
Tea	
No	2,397 (76.3%)
Yes	785 (24.7%)
Family history of cancer	
No	2,683 (84.3%)
Yes	499 (16.7%)
Total protein (g/L)	
≥35	3,096 (97.3%)
<35	86 (2.7%)
Albumin	
≥20	3,173 (99.7%)
<20	9 (0.3%)
Creatinine (μmol/L)	
≤115	3,098 (97.4%)
>115	84 (2.6%)
Total Bilirubin (μmol/L)	
≤34.2	3,110 (97.7%)
>34.2	72 (2.3%)
AST (U/L)	
≤40	2,852 (89.6%)
>40	330 (10.4%)
ALT (U/L)	
≤100	3,116 (97.9%)
>100	68 (2.1%)
Hemoglobin (g/L)	
≥110	2,416 (75.9%)
<110	766 (24.1%)
White Blood Cell (×10^9^/L)	
≤10	2,881 (90.5%)
>10	301 (9.5%)
Red Blood Cell (×10^12^/L)	
≥4.0	2,166 (68.0%)
<4.0	1,016 (32.0%)
Platelet (×10^9^/L)	
≥100	2,985 (93.8%)
<100	197 (6.25)
TNM stage	
I	258 (8.1%)
II	915 (28.8%)
III	1,279 (40.2%)
IV	730 (22.9%)
KPS score	
Independent	2,834 (89.1%)
Semi-Dependent	266 (8.4%)
Dependent	82 (2.6%)
PG-SGA	
Well-nourished	2,574 (80.9%)
Malnourished	608 (19.1%)
ESPEN	
Well-nourished	1,469 (46.2%)
Malnourished	1,713 (53.0%)
GLIM	
Well-nourished	2,550 (80.1%)
Malnourished	632 (19.9%)

^1^*n*(%). AST, Aspartate Aminotransferase; ALT, Alanine Aminotransferase; KPS, Karnofsky Performance Status; PG-SGA, Patient-Generated Subjective Global Assessment; ESPEN, European Society for Clinical Nutrition and Metabolism; GLIM, the Global Leadership Initiative on Malnutrition.

### 3.2 Analysis of discrepancies in nutritional diagnosis

To investigate the reasons for the significantly higher prevalence of malnutrition diagnosed by the ESPEN criteria (53.8%) compared to the PG-SGA (19.1%) and GLIM criteria (19.9%), we performed a subsequent analysis of discrepantly diagnosed patients. As shown in Table 2, among the 1,081 patients diagnosed as malnourished by ESPEN but well-nourished by GLIM, 255 patients (23.6%) were uniquely identified by ESPEN due to a body mass index (BMI) between 18.5 and 20 kg/m^2^ (and age <70 years). Furthermore, 182 patients (16.8%) had weight loss that met the ESPEN criterion of “>10% at any time” but did not fulfill the GLIM’s requirement for the weight loss to have occurred “beyond 6 months.” These findings indicate that the more inclusive BMI threshold and the broader time frame for weight loss in the ESPEN criteria are the primary drivers of its higher diagnosed prevalence.

**Table 2:** Characteristics of patients discrepantly diagnosed by ESPEN and GLIM criteria.

Characteristic	Total cohort (*N* = 3,182)	ESPEN-only malnourished (*n* = 1,081)	Both ESPEN and GLIM malnourished (*n* = 632)	*p*-value
Age <70 years, *n* (%)	2,120 (66.6)	758 (70.1)	290 (45.9)	<0.001
BMI for Patients <70 years, *n* (%)				
BMI < 18.5 (Meets both GLIM and ESPEN)	85 (2.7)	0 (0.0)	85 (13.4)	<0.001
BMI 18.5 - < 20 (Meets ESPEN but not GLIM)	255 (8.0)	255 (23.6)	0 (0.0)	255 (8.0)
Unintentional Weight Loss, *n* (%)				
Meets GLIM criteria (>5% in 6mo or >10% beyond 6mo)	900 (28.3)	268 (24.8)	632 (100.0)	<0.001
Meets only ESPEN criteria (>10% at any time, but beyond 6mo)	182 (5.7)	182 (16.8)	0 (0.0)	182 (5.7)
Fat-Free Mass Index (BIA), *n* (%)				
Reduced FFMI (ESPEN criterion 3)	732 (23.0)	263 (24.3)	469 (74.2)	<0.001
PG-SGA Physical Exam (Muscle Mass), *n* (%)				
Reduced Muscle Mass (GLIM phenotypic criterion)	950 (29.9)	318 (29.4)	632 (100.0)	<0.001
ESPEN Diagnostic Pathway, *n* (%)				
Diagnosed via Criterion 2 (Weight loss + BMI) only	Not Applicable	818 (75.7)	Not Applicable	Not Applicable
- Subgroup: Diagnosed due to BMI 18.5- < 20 (Age <70y)	Not Applicable	255 (23.6)	Not Applicable	Not Applicable
- Subgroup: Diagnosed due to “>10% at any time” WL	Not Applicable	182 (16.8)	Not Applicable	Not Applicable
Diagnosed via Criterion 3 (Weight loss + FFMI)	Not Applicable	263 (24.3)	Not Applicable	Not Applicable

The “ESPEN-only Malnourished” group comprises patients diagnosed as malnourished by ESPEN but well-nourished by GLIM (*n* = 1,713–632 = 1,081). *p*-values were calculated using the Chi-square test comparing the “ESPEN-only” group with the “Both ESPEN & GLIM” group. This table quantifies that the higher ESPEN prevalence is largely driven by its more inclusive BMI threshold (capturing patients with BMI 18.5- < 20) and its acceptance of weight loss that occurred “at any time”.

### 3.3 Comparative analysis of diagnostic value

**Figure 2:**
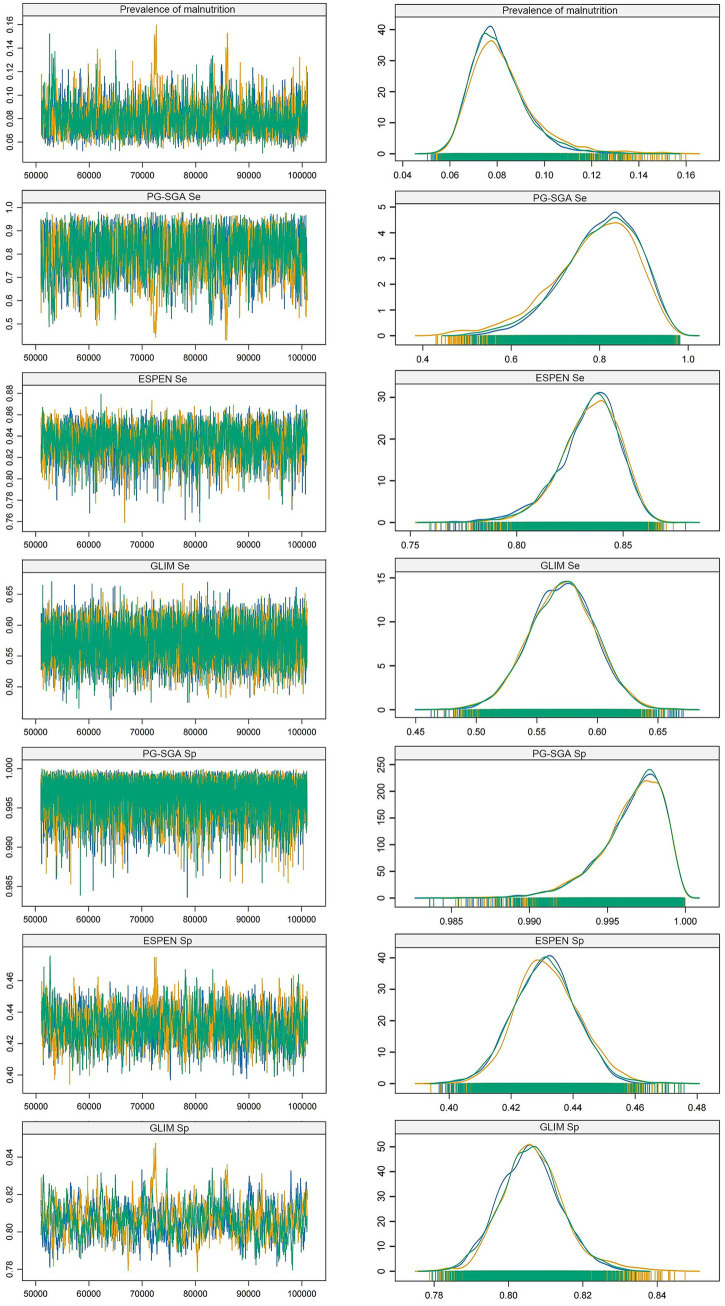
Trace plots and density plots of sensitivity and specificity of each tool. Se, Sensitivity; Sp, Specificity; PG-SGA, The patient-generated subjective global assessment; ESPEN, European Society for Clinical Nutrition and Metabolism; GLIM, the Global Leadership Initiative on Malnutrition.

We applied Bayesian principles to compare the diagnostic values of three nutritional assessment tools without establishing a reference standard. Through the analysis of trace plots, we found that the PG-SGA demonstrated both high sensitivity (0.80, 95% CrI: 0.61–0.94) and specificity (0.99, 95% CrI: 0.99–1.00); the ESPEN criteria exhibited high sensitivity (0.84, 95% CrI: 0.80–0.86) but low specificity (0.43, 95% CrI: 0.41–0.45), whereas the GLIM criteria showed low sensitivity (0.57, 95% CrI: 0.51–0.62) but high specificity (0.81, 95% CrI: 0.79–0.82). In addition, the density plots indicated a high degree of fit among the three Markov chains (Figure 2). Using the Gelman–Rubin test, we observed that the shrink factors for all diagnostic criteria approached 1.0 as the number of iterations increased (Supplementary material 2). These results suggest that all the diagnostic methods exhibit good convergence. It is noteworthy that the Bayesian latent class model employed here is designed to estimate sensitivity and specificity in the absence of a gold standard. Within this framework, traditional metrics that rely on a known gold standard (such as predictive values and likelihood ratios) are not straightforward to compute and their interpretation is less clear; therefore, they are not reported in this study.

### 3.4 Risk factor analysis

To identify the risk factors affecting the prognosis of patients with CRC, we performed univariate and multivariate Cox regression analyses. We found that age, alcohol consumption, creatinine, total bilirubin, white blood cell count, red blood cell count, TNM staging (stages III and IV), KPS score (semi-dependent grade), and malnutrition diagnosed by the GLIM criteria were independent risk factors influencing the patient’s prognosis (Table 3). Notably, among the nutritional assessment criteria, univariate Cox regression analysis suggested statistically significant associations between malnutrition diagnosed using the PG-SGA, ESPEN, and GLIM criteria and survival time. However, when incorporating all variables into a multivariable Cox regression analysis, only the association between malnutrition diagnosed using the PG-SGA and GLIM criteria and survival time remained statistically significant. Notably, the GLIM criteria yielded the highest HR values in both univariate and multivariable regression analyses (Univariate regression: HR = 1.42, 95% CI: 1.22–1.66; Multivariate regression: HR = 1.32, 95% CI: 1.11–1.56). We further conducted Cox regression subgroup analyses, adjusted by age, diabetes, hypertension, alcohol, TNM stage, KPS score, albumin, AST, hemoglobin, white blood cell counting and red blood cell counting to evaluate the correlation between nutritional status and overall survival risk (Table 4). We found that moderate and severe malnutrition identified by GLIM criteria were significantly correlated with survival time (Univariate regression: HR = 1.29, 95% CI: 1.06–1.58, *p* = 0.012; Multivariate regression: HR = 1.26, 95% CI: 1.02–1.54, *p* = 0.029). Meanwhile, severe malnutrition identified by PG-SGA also had a significant correlation with survival time (Univariate regression: HR = 1.81, 95% CI: 1.37–2.37, P<0.001; Multivariate regression: HR = 1.45, 95% CI: 1.10–1.93, *p* = 0.009), but moderate or suspected malnutrition identified by PG-SGA had no significant correlation (Univariate regression: HR = 1.06, 95% CI: 0.86–1.30, *p* = 0.610; Multivariate regression: HR = 1.04, 95% CI: 0.84–1.28, *p* = 0.750). The sensitivity analysis using the complete-case dataset yielded virtually identical distributions for all baseline characteristics and nearly identical hazard ratios with overlapping confidence intervals in the multivariate survival model, confirming the robustness of the primary findings to the method of handling missing data (Supplementary materials 3, 4).

**Table 3:** Univariate and multivariate analyses of factors influencing CRC patient survival (Cox regression).

Characteristic	Univariable			Multivariable		
	HR^1^	95% CI^1^	*p*-value	HR^1^	95% CI^1^	*p*-value
Sex						
Male	–	–		–	–	
Female	0.96	0.84, 1.11	0.588	0.86	0.72, 1.03	0.105
Age						
<65	–	–		–	–	
≥65	1.39	1.21, 1.61	<0.001	1.31	1.12, 1.52	<0.001
Diabetes						
No	–	–		–	–	
Yes	1.25	1.01, 1.55	0.039	1.15	0.92, 1.44	0.225
Hypertension						
No	–	–		–	–	
Yes	1.21	1.03, 1.42	0.021	1.14	0.96, 1.36	0.143
Coronary heart disease						
No	–	–		–	–	
Yes	0.99	0.10, 1.41	0.964	0.90	0.63,1.30	0.586
Smoking						
No	–	–		–	–	
Yes	1.02	0.89, 1.17	0.796	1.04	0.86, 1.24	0.705
Alcohol						
No	–	–		–	–	
Yes	0.84	0.70, 1.01	0.067	0.82	0.67, 1.01	0.059
Tea						
No	–	–		–	–	
Yes	1.07	0.91, 1.25	0.409	0.96	0.80, 1.14	0.611
Family history of cancer						
No	–	–		–	–	
Yes	0.97	0.80, 1.17	0.716	0.96	0.80, 1.16	0.689
Total protein(g/L)						
≥35	–	–		–	–	
<35	1.43	0.99, 2.06	0.056	1.37	0.94, 2.00	0.104
Albumin(g/L)						
≥20	–	–		–	–	
<20	2.83	1.18, 6.82	0.020	1.11	0.45, 2.75	0.824
Creatinine(μmol/L)						
≤115	–	–		–	–	
>115	1.95	1.37, 2.78	<0.001	1.61	1.11, 2.32	0.012
Total bilirubin(μmol/L)						
≤34.2	–	–		–	–	
>34.2	2.30	1.63, 3.25	<0.001	1.95	1.36, 2.80	<0.001
AST(U/L)						
≤40	–	–		–	–	
>40	1.59	1.30, 1.94	<0.001	1.25	1.00, 1.56	0.049
ALT(U/L)						
≤100	–	–		–	–	
>100	1.23	0.76,1.99	0.401	0.82	0.46, 1.44	0.580
Hemoglobin(g/L)						
>110	–	–		–	–	
≤110	1.52	1.31,1.76	<0.001	1.15	0.96, 1.36	0.123
White blood cell (×10^9^/L)						
≤10	–	–		–	–	
>10	1.82	1.50,2.21	<0.001	1.51	1.23, 1.86	<0.001
Red blood cell (×10^12^/L)						
≥4.0	–	–		–	–	
<4.0	1.70	1.48, 1.95	<0.001	1.29	1.09, 1.51	0.002
Platelet(×10^9^/L)						
≥100	–	–		–	–	
<100	1.27	0.98, 1.65	0.069	1.33	1.01,1.74	0.039
TNM stage						
I	–	–		–	–	
II	1.32	0.87, 2.00	0.189	1.20	0.79, 1.83	0.131
III	2.22	1.49, 3.29	<0.001	2.02	1.35, 3.02	<0.001
IV	7.80	5.27,11.53	<0.001	3.77	2.47, 5.76	<0.001
KPS score						
Independent	–	–		–	–	
Semi-Dependent	1.83	1.31, 2.55	<0.001	1.26	0.89, 1.80	0.194
Dependent	2.40	1.98, 2.91	<0.001	1.94	1.59, 2.37	<0.001
PG-SGA						
Well-nourished	–	–		–	–	
Malnourished	1.24	1.04, 1.48	0.016	1.21	1.01, 1.46	0.041
ESPEN						
Well-nourished	–	–		–	–	
Malnourished	1.26	1.08, 1.46	0.003	1.14	0.96, 1.36	0.132
GLIM						
Well-nourished	–	–		–	–	
Malnourished	1.42	1.22, 1.66	<0.001	1.32	1.11, 1.56	0.001

^1^HR = Hazard Ratio, CI = Confidence Interval. No. Obs. = 3,182; KPS, Karnofsky Performance Status; PG-SGA, Patient-Generated Subjective Global Assessment; ESPEN, European Society for Clinical Nutrition and Metabolism; GLIM, the Global Leadership Initiative on Malnutrition.

**Table 4:** Univariate and multivariate Cox regression analyses of PG-SGA and GLIM criteria for factors affecting CRC patient survival rates.

Characteristic	Univariable			Multivariable		
	HR^1^	95% CI^1^	*p*-value	HR^1^	95% CI^1^	*p*-value
PG-SGA						
Well-nourished	–	–		–	–	
Possibly or moderately malnourished	1.06	0.86, 1.30	0.610	1.04	0.84, 1.28	0.750
Severely malnourished	1.81	1.37, 2.37	<0.001	1.45	1.10, 1.93	0.009
GLIM						
Well-nourished	–	–		–	–	
Moderately malnourished	1.29	1.06, 1.58	0.012	1.26	1.02, 1.54	0.029
Severely malnourished	1.67	1.36, 2.05	<0.001	1.50	1.21, 1.85	<0.001

No. Obs. = 3,182; KPS, Karnofsky Performance Status; PG-SGA, Patient-Generated Subjective Global Assessment; GLIM, the Global Leadership Initiative on Malnutrition. Adjusted by age, diabetes, hypertension, alcohol, TNM stage, KPS score, albumin, AST, hemoglobin, white blood cell counting and red blood cell counting.

Meanwhile, we acknowledge that factors such as specific sites of metastasis, detailed treatment modalities (e.g., curative vs. palliative surgery), and the Charlson Comorbidity Index (CCI) are also important prognostic factors. However, due to the multicenter, retrospective nature of this study, there was substantial missing data for these variables. Their inclusion would have significantly reduced the sample size and introduced uncontrollable bias. Therefore, to ensure model stability and result reliability based on the maximum available sample size, these variables were not included in the final model. This limitation is further addressed in the Discussion.

### 3.5 Kaplan–Meier analysis

**Figure 3:**
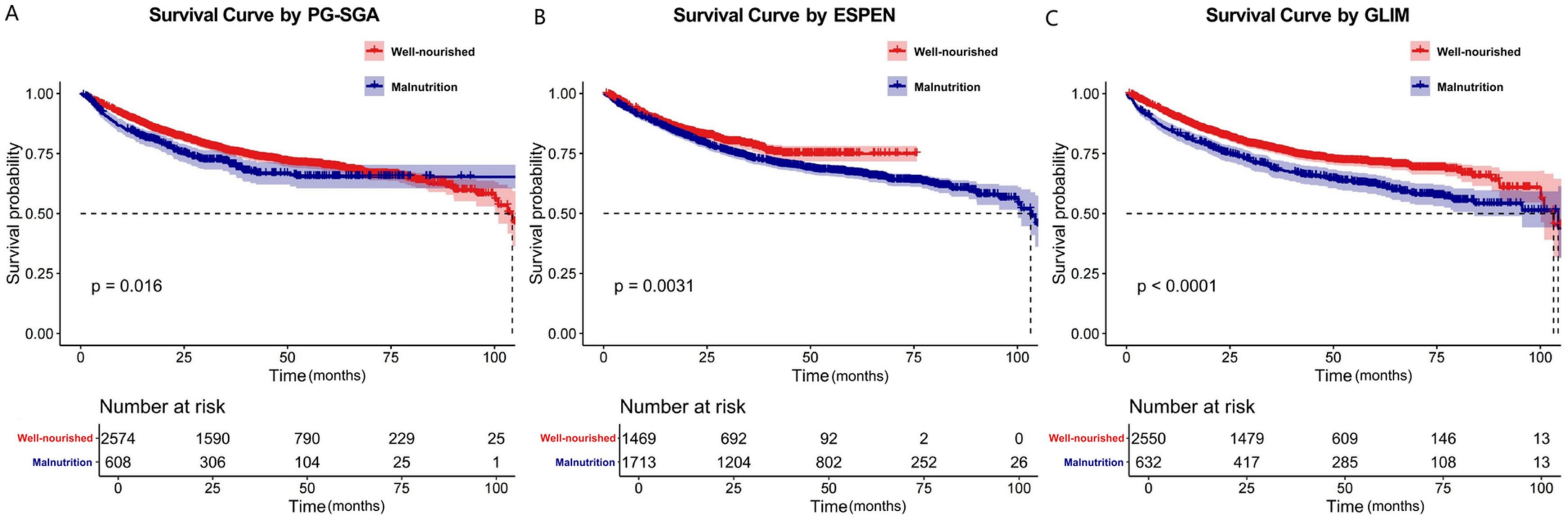
Kaplan–Meier curves for the overall survival of patients in different nutritional status groups diagnosed by the PG-SGA **(A)**, ESPEN **(B)**, or GLIM **(C)**. The overall survival rates between different nutritional status groups were analyzed and compared using Kaplan–Meier analysis and the log-rank test. PG-SGA, the Patient-Generated Subjective Global Assessment; ESPEN, European Society for Clinical Nutrition and Metabolism; GLIM, the Global Leadership Initiative on Malnutrition.

**Figure 4:**
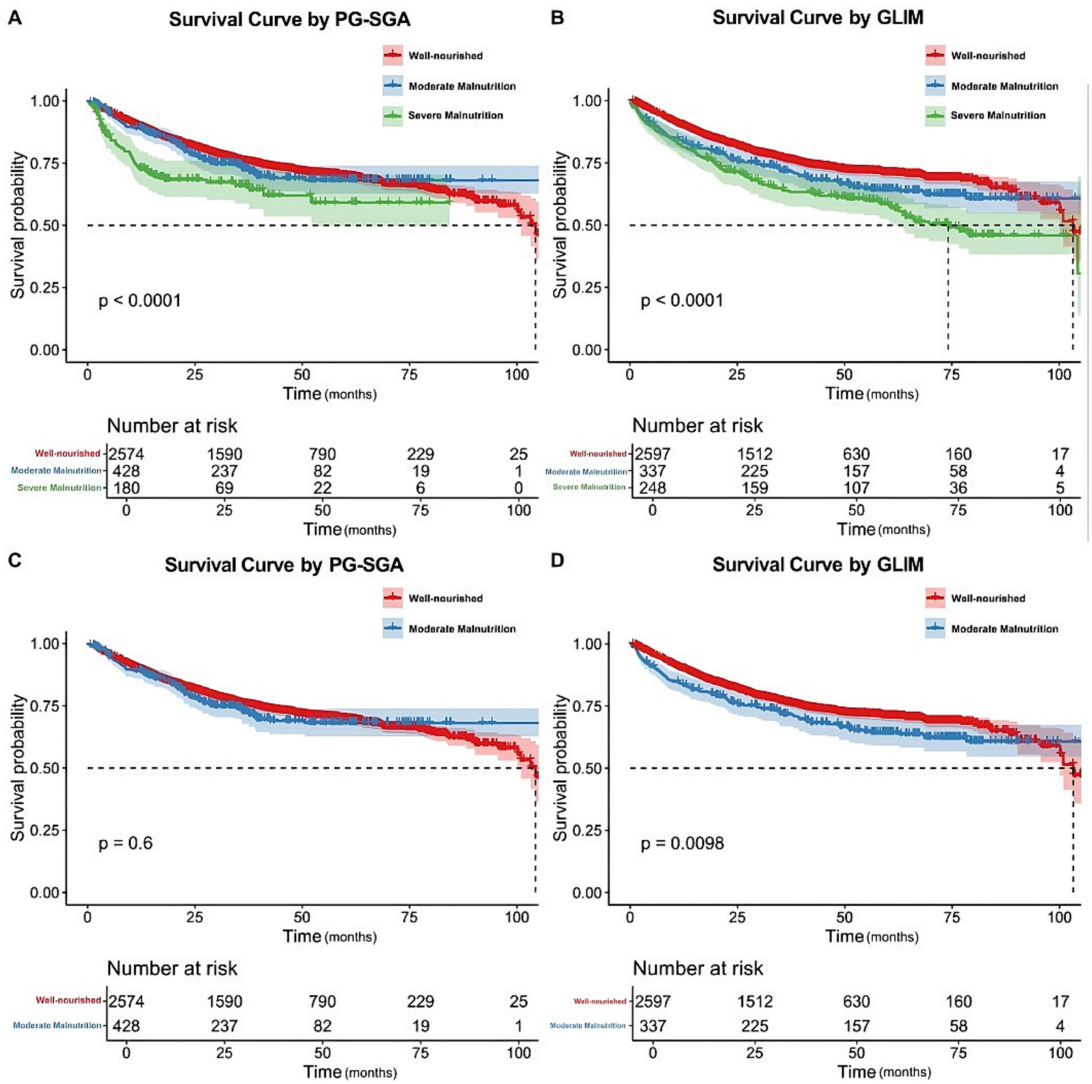
Kaplan–Meier curves for the overall survival of patients in different nutritional status groups diagnosed by the PG-SGA **(A–C)** and GLIM **(B–D)**. The overall survival rates between different nutritional status groups were analyzed and compared using Kaplan–Meier analysis and the log-rank test. PG-SGA, the Patient-Generated Subjective Global Assessment; ESPEN, European Society for Clinical Nutrition and Metabolism; GLIM, the Global Leadership Initiative on Malnutrition.

In univariate analysis, the PG-SGA, ESPEN, and GLIM criteria were found to be independent risk factors influencing the survival of patients with CRC. To further investigate these findings, we constructed Kaplan–Meier survival curves separately for each of the three nutritional assessment methods stratified by nutritional status. The analysis revealed statistically significant differences in survival between the well-nourished and malnourished groups as determined using the PG-SGA, ESPEN, and GLIM criteria (Figure 3). To further explore these results, we performed additional analyses in which we stratified patients into distinct subgroups according to their nutritional status, as assessed by each of the nutritional evaluation tools. We then separately plotted Kaplan–Meier survival curves for each subgroup (Figure 4). Our analysis revealed that the GLIM criteria, which classified patients into well-nourished, moderately malnourished, and severely malnourished groups, demonstrated a statistically significant difference in overall survival across these groups. Conversely, when using the PG-SGA, no statistically significant difference in survival was found between the well-nourished group and those categorized as possibly or moderately malnourished. This finding is consistent with the results of univariate and multivariate Cox regression analyses and their subgroup analyses. Given that the ESPEN criteria do not provide a means of classifying the severity of malnutrition, a subgroup analysis based on different malnutrition levels was not conducted for this assessment tool.

## 4 Discussion

The present study aimed to validate the diagnostic value of the PG-SGA, ESPEN, and GLIM criteria for malnutrition in patients with CRC, as well as their predictive value for prognosis. By applying Bayesian principles to compare the diagnostic performance and prognostic analysis of these three nutritional assessment tools, we gained new insights into the diagnosis of malnutrition in patients with CRC.

Our study uncovered a pivotal finding: a significant discordance in the prevalence of malnutrition diagnosed by the ESPEN, PG-SGA, and GLIM criteria in patients with colorectal cancer. Contrary to some previous studies (12, 13), the prevalence identified by the ESPEN criteria (53.8%) in our cohort was substantially higher than that by the GLIM (19.9%) and PG-SGA (19.1%) criteria. Crucially, the Bayesian analysis and subsequent diagnostic pathway analysis in our study (Table 2) provide a clear explanation for this discrepancy. The ESPEN criteria demonstrated high sensitivity, stemming from several of its more inclusive diagnostic thresholds. Firstly, for patients under 70 years, the BMI threshold for diagnosis when combined with weight loss is <20 kg/m^2^, compared to <18.5 kg/m^2^ in the GLIM criteria. Our data show that this led to the unique identification of a substantial proportion (23.6%) of patients with a BMI between 18.5 and 20 kg/m^2^ by ESPEN alone. Secondly, the ESPEN criterion accepting weight loss “>10% at any time,” unlike GLIM’s focus on weight loss “beyond 6 months,” captured more historical weight loss events, contributing to 16.8% of the unique ESPEN-only diagnoses.

Although the ESPEN criteria identified a large number of patients as malnourished, its association with overall survival did not reach statistical significance in the multivariable Cox regression analysis (HR = 1.14, 95% CI: 0.96–1.36, *p* = 0.132). This finding should not be interpreted as a refutation of the ESPEN criteria’s value but rather illuminates its potential different clinical positioning compared to GLIM/PG-SGA. Our Bayesian analysis characterized ESPEN as a highly sensitive tool, whose design philosophy favors ‘casting a wide net’ to maximize the identification of patients with any nutritional risk. However, this high sensitivity likely results in a more heterogeneous diagnosed population. As illustrated in Table 2, the ESPEN-defined cohort includes a substantial number of patients identified solely due to mildly low BMI or historical weight loss. The nutritional compromise in these patients may be less severe, and their survival prognosis is likely more dominantly driven by stronger factors such as tumor stage and treatment tolerance, thereby ‘diluting’ the independent effect of nutritional status. This pattern echoes findings in predictive model development across oncology, where highly sensitive screening tools often identify broader risk spectra (27, 28). In contrast, GLIM and PG-SGA, with their higher specificity, more precisely target a patient subset with more severe phenotypic criteria (e.g., low BMI, recent significant weight loss), who are at a substantially higher risk of mortality, thus demonstrating a clearer independent association with survival outcomes.

Consequently, the clinical utility of the ESPEN criteria may lie primarily in early screening and comprehensive risk identification, suitable for guiding broad nutritional support and intervention to prevent further deterioration. Conversely, the GLIM criteria excel in prognostication, risk stratification, and identifying severely affected patients requiring intensive nutritional therapy. Our findings suggest that the choice of nutritional assessment tool in clinical practice and research should be aligned with the specific objective—whether it is widespread screening or prognosis evaluation.

In the comparison of diagnostic values, we found that the PG-SGA remained a diagnostic method with high sensitivity and specificity. In the prognostic study section, we observed that the PG-SGA, ESPEN, and GLIM criteria effectively differentiated the survival periods of well-nourished and malnourished patients. This aligns with methodological principles in prognostic model development, where specificity often enhances predictive accuracy for severe outcomes (29). Moreover, the GLIM criteria showed a superior ability to distinguish survival periods among subgroups of patients with different nutritional statuses.

Despite being introduced nearly 30 years ago, our study demonstrates that the PG-SGA remains a highly valuable nutritional evaluation method for diagnosing malnutrition in patients with CRC. Although the ESPEN and GLIM criteria were introduced only recently, we found in our study that the ESPEN criteria had low specificity (0.43, 95% CrI: 0.41–0.45), whereas the GLIM criteria exhibited low sensitivity (0.57, 95% CrI: 0.51–0.62) (Figure 2). Overall, the PG-SGA demonstrated the highest diagnostic performance.

Since there is no established “gold standard” for diagnosing malnutrition, and given its dynamically changing prevalence in real-world scenarios, we chose not to use any single diagnostic tool as a reference standard for comparing other criteria. Instead, we adopted a gold-standard-free comparison method based on Bayesian principles. This approach represents the major strength of this study.

Prior research has similarly employed Bayesian principles to compare different nutritional assessment and screening tools (30, 31). Nevertheless, it is important to highlight the overlap in the application of nutritional screening tools and malnutrition diagnostic methods, a topic that merits further exploration in future studies.

Our findings, considered alongside recent advances in predictive modeling (27, 32), suggest that future nutritional assessment might benefit from integrating both biological and psychosocial dimensions. As demonstrated in cancer care research, the intersection of physiological and psychological factors significantly influences patient outcomes (32).

In the subgroup analysis, the GLIM criteria demonstrated its potential for prognosis prediction, with its diagnosis showing a statistically significant association with survival outcomes. However, it is important to note that this study did not directly compare statistical metrics of prognostic discrimination (e.g., C-index) between tools. Therefore, the ‘superiority’ of GLIM in prognostication is primarily reflected in the consistent and significant risk association afforded by its high specificity, rather than a direct comparison of model discrimination. Simultaneously, we acknowledge that the operational complexity of the PG-SGA, as a comprehensive assessment tool, may limit its widespread use for routine clinical diagnosis, though the detailed information it provides is invaluable for formulating individualized nutritional support plans.

Combining these findings with our study results, we propose that in future clinical practice, the PG-SGA should continue to be used as a tool for diagnosing malnutrition owing to its overall diagnostic efficacy. However, when integrating nutritional assessment outcomes to predict patient prognosis, the GLIM criteria may be considered a more suitable choice. This dual approach leverages the strengths of both tools: the broad applicability and accuracy of the PG-SGA for diagnosis and the refined prognostic predictive power of the GLIM criteria for stratifying patient outcomes.

Our study has certain limitations. First, all patients in this study were recruited from tertiary hospitals in China, which may limit the generalizability of our findings to patients in community, rural settings, or other geographical regions. External validation in diverse healthcare settings and populations is needed to confirm our conclusions. Second, this study is a retrospective analysis and therefore cannot establish causality, and may be subject to selection and information biases. Future prospective studies are necessary to validate our findings. Finally, a sensitivity analysis confirmed that our primary survival conclusions remained unchanged despite the use of mode substitution for minimal missing baseline data.

Future research should address these limitations through prospective studies with broader patient populations and the development of new nutritional diagnostic criteria. Such advancements are crucial for improving the diagnosis and prognosis of malnutrition in clinical practice.

## 5 Conclusion

In conclusion, the ESPEN, GLIM, and PG-SGA criteria have distinct emphases in nutritional assessment for CRC patients. The ESPEN criteria, with high sensitivity, is more suitable for large-scale clinical screening to achieve early and broad identification of patients at risk. The GLIM criteria, with high specificity and a significant association with survival outcomes, shows important value for prognostication and risk stratification. The PG-SGA, due to its comprehensive nature, remains crucial in settings requiring in-depth assessment for complex nutritional therapy planning. Future research should focus on directly comparing the predictive performance of these tools using more advanced statistical metrics (e.g., C-index, NRI) and exploring the feasibility and benefit of integrating them in clinical workflows (e.g., using ESPEN for initial screening, followed by GLIM/PG-SGA for severity assessment and prognosis in screen-positive patients), thereby establishing efficient nutritional care pathways.

## Data Availability

The datasets presented in this article are not readily available because access to the raw data is granted only upon submission of a formal application to the INSCOC Project Committee. Requests to access the datasets should be directed to sch16@zzu.edu.cn.

## Footnotes

1
https://www.chictr.org.cn/showproj.html?proj=31813

2 version 3.6.3; http://www.r-project.org/.
