# Supplementary material for: Comparative performance of the patient-generated subjective global assessment, European Society for Clinical Nutrition and Metabolism criteria, and Global Leadership Initiative on Malnutrition criteria in patients with colorectal cancer: a multicenter study utilizing Bayesian inference
Source: Front Nutr. 2026 Feb 16;12:1671154. doi: 10.3389/fnut.2025.1671154 (PMC12950724; doi:10.3389/fnut.2025.1671154)
Supplement: Supplementary file 1 [file Image_1.pdf]

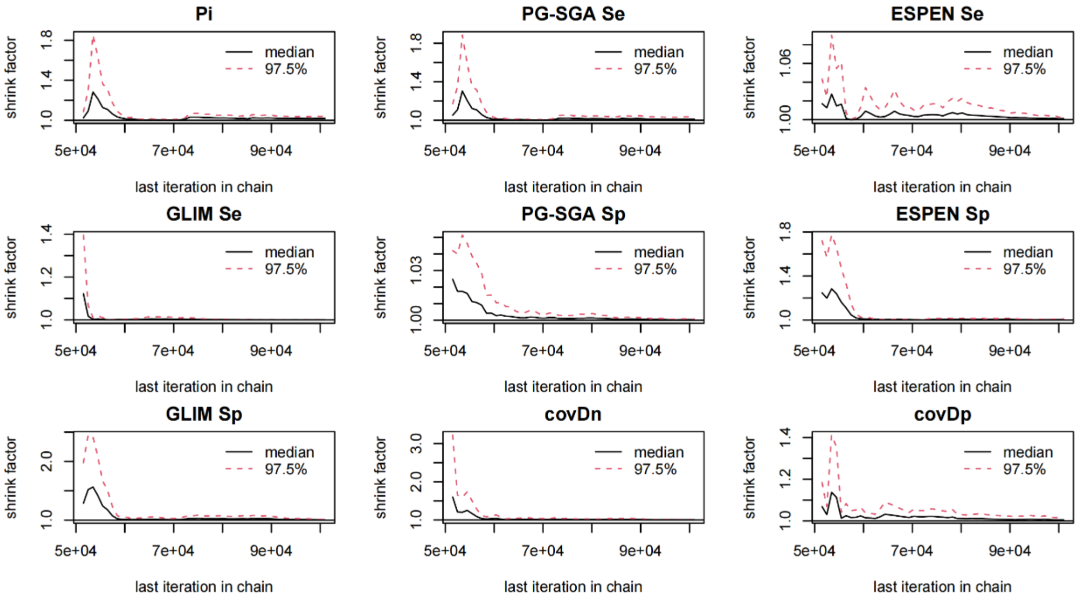

Supplementary Figure 1. Gelman-Rubin test for the prevalence of malnutrition and tool sensitivity and specificity. Se, sensitivity; Sp, specificity; covDp and covDn, covariance of PG-SGA, ESPEN criteria and GLIM criteria among the malnourished or normally nourished subject, respectively. PG-SGA, the Patient-Generated Subjective Global Assessment; ESPEN, European Society for Clinical Nutrition and Metabolism; GLIM, the Global Leadership Initiative on Malnutrition.
