# Supplementary material for: Comparative performance of the patient-generated subjective global assessment, European Society for Clinical Nutrition and Metabolism criteria, and Global Leadership Initiative on Malnutrition criteria in patients with colorectal cancer: a multicenter study utilizing Bayesian inference
Source: Front Nutr. 2026 Feb 16;12:1671154. doi: 10.3389/fnut.2025.1671154 (PMC12950724; doi:10.3389/fnut.2025.1671154)
Supplement: Supplementary file 2 [file Table_1.docx]

**Supplementary Material 1. Simplified Explanation and Translation of BLCM**

In the field of malnutrition diagnosis, the absence of a universally recognized gold standard, coupled with the dynamic nature of malnutrition prevalence, poses significant challenges. To address these issues, this study employs an innovative statistical approach known as the Bayesian Latent Class Model (BLCM). This model allows for a systematic comparison of the diagnostic efficacies of three commonly used nutritional assessment tools—PG-SGA, ESPEN criteria, and GLIM criteria—in the absence of a gold standard.

The BLCM offers a key advantage by integrating prior knowledge with current research data to optimize the estimation of diagnostic tools' sensitivity and specificity through a Bayesian statistical framework.

The fundamental principles of BLCM involve several crucial steps. First, prior distributions are constructed based on historical data or expert consensus, reflecting the historical performance of diagnostic tools in similar populations. Second, the prior distributions are updated using the observed data from the current study through Bayes' theorem to obtain posterior distributions. Finally, the latest performance metrics of the diagnostic tools, including sensitivity, specificity, and their credible intervals, are derived from the posterior distributions.

This approach not only effectively overcomes the limitations posed by the absence of a gold standard but also leverages existing research information to enhance the reliability and accuracy of the estimation results^[1,2]^.

By applying the BLCM, this study provides an objective comparison of different diagnostic criteria within a unified statistical framework. The following sections offer a detailed explanation of this method.

**1.Model Setup**

1. Assessment Tools and Sample Characteristics: The study included K = 3 commonly used clinical nutrition assessment tools: PG-SGA, ESPEN criteria, and GLIM criteria. These tools were used to evaluate the nutrition status of N = 3,182 CRC patients.
2. Data Structure of Test Results: For the i -th subject, the test result is a binary vector Ti = (Ti1, Ti2, Ti3), where Tik ∈ {0, 1}. Tik = 1 indicates a positive result (malnutrition exists), while Tik = 0 indicates a negative result (no malnutrition).
3. Mathematical Expression of Latent True State: Di represents the latent true nutrition status of the i -th subject. Di = 1 means actual nutrition risk or malnutrition, and Di = 0 means no nutrition risk. This latent state can be inferred through the joint distribution of multiple test results.
4. Parameterization of Prevalence: The parameter π represents the prevalence of actual malnutrition in the population, π = P (Di = 1), reflecting the real distribution of nutrition risk in the study population.
5. Quantification of Diagnostic Performance: For the k -th test tool, diagnostic performance is quantified by sensitivity and specificity. Sensitivity (Sek) is the probability of correctly identifying positives among patients with actual nutrition risk, Sek = P (Tik = 1 | Di = 1). Specificity (Spk) is the probability of correctly identifying negatives among patients without actual nutrition risk, Spk = P (Tik = 0 | Di = 0).

**2. Conditional Dependence**

ESPEN and GLIM criteria were both assessed within 48 hours of admission and share some core indicators (e.g., BMI, non - voluntary weight loss), which may lead to conditional dependence between the two diagnostic tools under the true nutrition status. To quantify this correlation, covariance parameters covDp and covDn were introduced to characterize the conditional dependence strength between the two criteria under malnutrition (Di = 1) and normal nutrition (Di = 0) states, respectively.

**3. Joint Probability**

In the Bayesian latent class model framework, joint probability characterizes the distribution of multiple diagnostic tool result combinations. With three nutrition assessment tools (PG - SGA, ESPEN, and GLIM criteria), there are 2³ = 8 possible combination patterns of binary diagnostic results. A conditional probability matrix was constructed to analyze the association between different diagnostic result combinations and the true nutrition status. Taking the typical case where all three tools are positive (*T_i1_*=1, *T_i2_*=1, *T_i3_*=1) as an example, the joint probabilities under true malnutrition and normal nutrition states are expressed as follows:

True malnutrition state (Di=1):

P ( Ti1 =1, Ti2 =1, Ti3 =1|Di = 1) = Se₁(Se₂Se₃ + covDp)

True normal nutrition state (Di=0):

P ( Ti1 =1, Ti2 =1, Ti3 =1|Di = 0) = (1 - Sp₁)[(1 - Sp₂)(1 - Sp₃) + covDn]

**4. Likelihood Function and Parameter Estimation**

In the Bayesian latent class model, the likelihood function connects the observed data with model parameters, quantifying the probability of observing the data given the parameter values. A likelihood function for multi - tool joint diagnosis was constructed to accurately estimate model parameters. Let θ = (π, Se₁, Se₂, Se₃, Sp₁, Sp₂, Sp₃, covDp, covDn) be the vector of parameters to be estimated. The observed data N = (N₁, N₂, ..., N₈) represents the frequency distribution of the eight diagnostic result combinations. The likelihood function can be expressed as follows:

L = L (N|θ) ∝ ∏ₘ=1⁸ [P (T₁ = t₁, T₂ = t₂, T₃ = t₃|D) ]^Nm

where m represents the eight diagnostic result combinations, and t₁, t₂, t₃ ∈ {0, 1}.

**5. Prior Distribution and Justification**

In the Bayesian statistical framework, the prior distribution incorporates existing knowledge before observing the current data. Given the absence of a gold standard, we utilized informative prior distributions to stabilize the Bayesian Latent Class Model (BLCM) and improve the identifiability of parameters, a common and recommended practice in such diagnostic accuracy studies [1, 2].

The selection of priors was based on a comprehensive and systematic review of 34 previous studies [3-36] that assessed the performance of PG-SGA, ESPEN, or GLIM criteria in various adult cancer populations. For each parameter, we synthesized the reported performance estimates from the literature. We then modeled the prior distributions using Beta distributions, which are conjugate priors for binomial proportions. The parameters of the Beta distribution (α and β) were chosen to reflect the central tendency (mean) and uncertainty (variance) of the historical data. The BetaBuster software (version 1.0) was used to determine the parameters (α and β) of the Beta distributions .

Supplementary Table 1 details the prior distributions for each parameter, their corresponding means and 95% intervals, and the key literature supporting these choices. For instance:

The prior for PG-SGA Sensitivity was set to Beta (32.166, 4.463), corresponding to a mean sensitivity of 0.88. This strong prior is supported by multiple studies in cancer populations synthetically reporting high sensitivity for PG-SGA [4-14].

The prior for PG-SGA Specificity was set to Beta (9.200, 2.800), with a mean of 0.77. This reflects the literature where PG-SGA, while specific, may occasionally misclassify well-nourished patients with high symptom burden [4-14].

| Supplementary Table 1. Justification of Prior Distributions for the Bayesian Latent Class Model   \| Parameter \| Tool \| Beta(a,b) \| Mean (95% Interval) \| Rationale and Key Supporting Literature \| \| --- \| --- \| --- \| --- \| --- \| \| Prevalence \|  \| (6.168, 5.349) \| 0.54 (0.31-0.75) \| Based on the wide range of reported malnutrition prevalence in colorectal cancer studies from our literature review. \| \| Sensitivity \| PG-SGA \| (32.166, 4.463) \| 0.8782 (0.7562, 0.9615) \| Synthetically high sensitivity reported in multiple oncology studies [4-14]. \| \|  \| ESPEN \| (37.984, 31.259) \| 0.5486 (0.4312, 0.6632) \| Literature suggests often moderate sensitivity, highly dependent on the population and applied criteria [3, 8, 15, 16]. \| \|  \| GLIM \| (67.696, 40.171) \| 0.6276 (0.5347, 0.716) \| Emerging body of evidence indicates moderate to high sensitivity, though often lower than PG-SGA [3, 17, 18-36]. \| \| Specificity \| PG-SGA \| (9.200, 2.800) \| 0.7667 (0.502, 0.9483) \| High specificity is commonly reported, though not perfect [4-14]. \| \|  \| ESPEN \| (102.396, 3.069) \| 0.9709 (0.9315, 0.9937) \| ESPEN criteria, relying on objective measures, are frequently reported to have very high specificity [3, 8, 15, 16]. \| \|  \| GLIM \| (228.155, 41.086) \| 0.8474 (0.8022, 0.8877) \| GLIM specificity is generally high, with estimates clustering around this prior mean across multiple validation studies [3, 17, 18-36]. \| |
| --- | --- | --- | --- | --- | --- | --- | --- | --- | --- | --- | --- | --- | --- | --- | --- | --- | --- | --- | --- | --- | --- | --- | --- | --- | --- | --- | --- | --- | --- | --- | --- | --- | --- | --- | --- | --- | --- | --- | --- | --- |

Note: PG-SGA, Patient-Generated Subjective Global Assessment; ESPEN, European Society for Clinical Nutrition and Metabolism criteria; GLIM, Global Leadership Initiative on Malnutrition criteria

**6. Posterior Distribution and Markov Chain Monte Carlo (MCMC) Method**

The posterior distribution in Bayesian statistical inference is the updated parameter distribution obtained by combining the prior distribution with observed data. It reflects the correction of the parameter probability distribution after incorporating new evidence. MCMC methods were employed to estimate the posterior distribution. The process involved the following steps:

1. Fitting the Bayesian model using MCMC algorithms to calculate the posterior distribution of target parameters.
2. Implementing MCMC calculations with the "rjags" package in the R language environment. Three independent Markov chains were run in parallel, each performing 50,000 iterations. The first 10,000 iterations were discarded as burn - in, and thinning was applied to the remaining 40,000 iterations, with one sample drawn every 10 iterations to improve independence and convergence.
3. Assessing MCMC convergence through trace plots and Gelman - Rubin diagnostic plots. All parameters achieved convergence, with Gelman-Rubin diagnostic (R̂) values ≤ 1.01, well below the recommended threshold of 1.1.
4. After confirming convergence, the mean of the posterior distribution was used as the point estimate, and the 95% credible interval (CrI) was reported to quantify parameter uncertainty.

**7. Sensitivity Analysis of Prior Distributions**

To directly address the potential concern that our results are overly dependent on the chosen informative priors, we conducted a comprehensive sensitivity analysis. We re-fitted the BLCM under three distinct prior scenarios:

Scenario 1 (Original Informative Priors): The priors as described in Section 5 and Supplementary Table 1.

Scenario 2 (Vague Priors): We used non-informative Beta (1,1) priors for all parameters (prevalence, sensitivities, and specificities). This distribution is uniform over (0,1) and represents a state of minimal prior knowledge, letting the data dominate the posterior.

The posterior estimates for sensitivity and specificity under these two scenarios are presented in Supplementary Table 2. The key findings are:

When non-informative Beta (1,1) priors were applied, the model yielded posterior estimates for PG-SGA that were clinically implausible, with a sensitivity of 0.125 (95% CrI: 0.101-0.152) and a specificity of 0.999 (95% CrI: 0.996-1.000). This combination contradicts the established high sensitivity of PG-SGA reported in extensive literature and is inconsistent with the observed prevalence of malnutrition identified by PG-SGA in our own dataset (19.1%). Furthermore, the 95% credible intervals for the covariance parameters (covDp and covDn) included zero, indicating failure to reliably estimate the conditional dependence between ESPEN and GLIM criteria under this prior specification. These results demonstrate that in the absence of any prior knowledge, the BLCM can produce unstable and clinically unreliable estimates, a known limitation of such complex models when applied to data without a gold standard. This underscores the necessity of incorporating well-justified informative priors to stabilize the model and yield biologically and clinically interpretable results.

| Supplementary Table 2. Results of Sensitivity Analysis for Bayesian Latent Class Model under Different Prior Scenarios   \| Tool \| Parameter \| Scenario 1: Original Priors \| Scenario 2: Vague Priors [Beta(1,1)] \| \| --- \| --- \| --- \| --- \| \| PG-SGA \| Sensitivity \| 0.80 (0.61-0.94) \| 0.13 (0.10-0.15) \| \|  \| Specificity \| 0.99 (0.99-1.00) \| 0.99 (0.99-1.00) \| \| ESPEN \| Sensitivity \| 0.84 (0.80-0.86) \| 0.99 (0.98-0.99) \| \|  \| Specificity \| 0.61 (0.58-0.63) \| 0.77 (0.66-0.93) \| \| GLIM \| Sensitivity \| 0.59 (0.56-0.61) \| 0.45 (0.38-0.53) \| \|  \| Specificity \| 0.81 (0.79-0.82) \| 0.99 (0.99-1.00) \| |
| --- | --- | --- | --- | --- | --- | --- | --- | --- | --- | --- | --- | --- | --- | --- | --- | --- | --- | --- | --- | --- | --- | --- | --- | --- | --- | --- | --- | --- |

Values presented as Posterior Mean (95% Credible Interval).

**8. Posterior Predictive Check**

To validate the model's goodness-of-fit, we performed a posterior predictive check. This involved comparing the observed frequencies of the eight diagnostic result combinations with the frequencies predicted by our fitted Bayesian model. The results showed a close alignment between the observed and predicted frequencies, with a Bayesian p-value of 0.52. This indicates no significant discrepancy between the model and the observed data, supporting the adequacy of our model fit.

**References**

1. Dendukuri N, Joseph L. Bayesian approaches to modeling the conditional dependence between multiple diagnostic tests[J]. Biometrics, 2001,57(1):158-167.
2. Joseph L, Gyorkos T W, Coupal L. Bayesian estimation of disease prevalence and the parameters of diagnostic tests in the absence of a gold standard[J]. Am J Epidemiol, 1995,141(3):263-272.
3. Poulter S, Steer B, Baguley B, et al. Comparison of the GLIM, ESPEN and ICD-10 Criteria to Diagnose Malnutrition and Predict 30-Day Outcomes: An Observational Study in an Oncology Population[J]. Nutrients, 2021,13(8).
4. Bauer J, Capra S, Ferguson M. Use of the scored Patient-Generated Subjective Global Assessment (PG-SGA) as a nutrition assessment tool in patients with cancer[J]. Eur J Clin Nutr, 2002,56(8):779-785.
5. Gabrielson D K, Scaffidi D, Leung E, et al. Use of an Abridged Scored Patient-Generated Subjective Global Assessment (abPG-SGA) as a Nutritional Screening Tool for Cancer Patients in an Outpatient Setting[J]. NUTRITION AND CANCER-AN INTERNATIONAL JOURNAL, 2013,65(2):234-239.
6. Vázquez De La Torre M J, Stein K, Vásquez Garibay E M, et al. Patient-Generated Subjective Global Assessment of nutritional status in pediatric patients with recent cancer diagnosis[J]. Nutr Hosp, 2017,34(5):1050-1058.
7. Du H, Liu B, Xie Y, et al. Comparison of different methods for nutrition assessment in patients with tumors[J]. ONCOLOGY LETTERS, 2017,14(1):165-170.
8. Ding H, Dou S, Ling Y, et al. Longitudinal Body Composition Changes and the Importance of Fat-Free Mass Index in Locally Advanced Nasopharyngeal Carcinoma Patients Undergoing Concurrent Chemoradiotherapy[J]. Integr Cancer Ther, 2018,17(4):1125-1131.
9. Zhang Y H, Xie F Y, Chen Y W, et al. Evaluating the Nutritional Status of Oncology Patientsand Its Association with Quality of Life[J]. BIOMEDICAL AND ENVIRONMENTAL SCIENCES, 2018,31(9):637-644.
10. Yang D, Zheng Z, Zhao Y, et al. Patient-generated subjective global assessment versus nutritional risk screening 2002 for gastric cancer in Chinese patients[J]. Future Oncol, 2020,16(3):4475-4483.
11. Dong W, Liu X, Zhu S, et al. Selection and optimization of nutritional risk screening tools for esophageal cancer patients in China[J]. NUTRITION RESEARCH AND PRACTICE, 2020,14(1):20-24.
12. Zhang Z, Wan Z, Zhu Y, et al. Prevalence of malnutrition comparing NRS2002, MUST, and PG-SGA with the GLIM criteria in adults with cancer: A multi-center study[J]. NUTRITION, 2021,83.
13. Tian M, Fu H, Du J. Application value of NRS2002 and PG-SGA in nutritional assessment for patients with cervical cancer surgery[J]. AMERICAN JOURNAL OF TRANSLATIONAL RESEARCH, 2021,13(6):7186-7192.
14. Tan S, Jiang J, Qiu L, et al. Prevalence of Malnutrition in Patients with Hepatocellular Carcinoma: A Comparative Study of GLIM Criteria, NRS2002, and PG-SGA, and Identification of Independent Risk Factors[J]. NUTRITION AND CANCER-AN INTERNATIONAL JOURNAL, 2024,76(4):335-344.
15. de Araujo B E, Kowalski V, Leites G M, et al. AND-ASPEN and ESPEN consensus, and GLIM criteria for malnutrition identification in AECOPD patients: a longitudinal study comparing concurrent and predictive validity[J]. EUROPEAN JOURNAL OF CLINICAL NUTRITION, 2022,76(5):685-692.
16. Ren S, Zhu M, Zhang K, et al. Machine Learning-Based Prediction of In-Hospital Complications in Elderly Patients Using GLIM-, SGA-, and ESPEN 2015-Diagnosed Malnutrition as a Factor[J]. NUTRIENTS, 2022,14(15).
17. De Groot L M, Lee G, Ackerie A, et al. Malnutrition Screening and Assessment in the Cancer Care Ambulatory Setting: Mortality Predictability and Validity of the Patient-Generated Subjective Global Assessment Short form (PG-SGA SF) and the GLIM Criteria[J]. Nutrients, 2020,12(8).
18. Qin L, Tian Q, Zhu W, et al. The Validity of the GLIM Criteria for Malnutrition in Hospitalized Patients with Gastric Cancer[J]. Nutr Cancer, 2021,73(11-12):2732-2739.
19. Wang Y, Chen X, Wang Y, et al. Body Composition Measurement Improved Performance of GLIM Criteria in Diagnosing Malnutrition Compared to PG-SGA in Ambulatory Cancer Patients: A Prospective Cross-Sectional Study[J]. Nutrients, 2021,13(8).
20. Zhang K, Tang M, Fu Z, et al. Global Leadership Initiative on Malnutrition criteria as a nutrition assessment tool for patients with cancer[J]. NUTRITION, 2021,91-92.
21. Henriksen C, Paur I, Pedersen A, et al. Agreement between GLIM and PG-SGA for diagnosis of malnutrition depends on the screening tool used in GLIM[J]. Clin Nutr, 2022,41(2):329-336.
22. Orell H K, Pohju A K, Osterlund P, et al. GLIM in diagnosing malnutrition and predicting outcome in ambulatory patients with head and neck cancer[J]. FRONTIERS IN NUTRITION, 2022,9.
23. Tan S, Wang J, Zhou F, et al. Validation of GLIM malnutrition criteria in cancer patients undergoing major abdominal surgery: A large-scale prospective study[J]. CLINICAL NUTRITION, 2022,41(3):599-609.
24. Da Silva Couto A, Gonzalez M C, Martucci R B, et al. Predictive validity of GLIM malnutrition diagnosis in patients with colorectal cancer[J]. JPEN J Parenter Enteral Nutr, 2023,47(3):420-428.
25. Huo Z, Chong F, Yin L, et al. Comparison of the performance of the GLIM criteria, PG-SGA and mPG-SGA in diagnosing malnutrition and predicting survival among lung cancer patients: A multicenter study[J]. Clin Nutr, 2023,42(6):1048-1058.
26. Ozorio G A, Ribeiro L M K, Santos B C, et al. Exploring the use of the GLIM criteria to diagnose malnutrition in cancer inpatients[J]. Nutrition, 2023,116:112195.
27. Crestani M S, Stefani G P, Scott L M, et al. Accuracy of the GLIM Criteria and SGA Compared to PG-SGA for the Diagnosis of Malnutrition and Its Impact on Prolonged Hospitalization: A Prospective Study in Patients with Cancer[J]. NUTRITION AND CANCER-AN INTERNATIONAL JOURNAL, 2023,75(4):1177-1188.
28. Zou Y, Xu H, Lyu Q, et al. Malnutrition diagnosed by GLIM criteria better predicts long-term outcomes for patients with non-Hodgkin's lymphoma: A prospective multicenter cohort study[J]. HEMATOLOGICAL ONCOLOGY, 2023,41(3):371-379.
29. Liu Y, Kang J, Qi Z, et al. Comparison of GLIM and PG-SGA for predicting clinical outcomes of patients with esophageal squamous carcinoma resection[J]. NUTRICION HOSPITALARIA, 2023,40(3):574-582.
30. Solon L A, Gomes K P, Da Luz M C L, et al. Comparison between GLIM and PG-SGA methods in the nutritional assessment of hospitalized oncological patients[J]. REVISTA DE NUTRICAO-BRAZILIAN JOURNAL OF NUTRITION, 2023,36.
31. Wang Y, Liu Z, Zhang H, et al. Evaluation of different screening tools as the first step of the GLIM framework: A cross-sectional study of Chinese cancer patients in an outpatient setting[J]. Nutr Clin Pract, 2024,39(3):702-713.
32. Wang J, Xu Q, Xie H, et al. Comparison of the Global Leadership Initiative on Malnutrition and the Patient-Generated Subjective Global Assessment for diagnosing malnutrition in patients undergoing surgery for hepatobiliary and pancreatic malignancies[J]. NUTRICION HOSPITALARIA, 2024,41(4):835-842.
33. Fu L, Xu X, Zhang Y, et al. Agreements between the Global Leadership Initiative on Malnutrition using left calf circumference as criterion for reduced muscle mass and the Patient-Generated Subjective Global Assessment, and the Global Leadership Initiative on Malnutrition using the appendicular skeletal muscle index for the diagnosis of malnutrition in gastric cancer patients[J]. NUTRICION HOSPITALARIA, 2024,41(4):824-834.
34. Gersely G D, Klein R C M, Da Rocha G D G V, et al. GLIM criteria validation and reliability in critically ill patients with cancer: A prospective study[J]. JOURNAL OF PARENTERAL AND ENTERAL NUTRITION, 2024,48(6):726-734.
35. Zhou L, Yu D, Ma B, et al. Feasibility of substituting handgrip strength for muscle mass as a constituent standard in the Global Leadership Initiative on Malnutrition for diagnosing malnutrition in patients with gastrointestinal cancers[J]. Nutrition, 2021,84:111044.
36. Santos A L S D, Santos B C, Frazao L N, et al. Validity of the GLIM criteria for the diagnosis of malnutrition in patients with colorectal cancer: A multicenter study on the diagnostic performance of different indicators of reduced muscle mass and disease severity[J]. NUTRITION, 2024,119.
