# Supplementary material for: Comparative performance of the patient-generated subjective global assessment, European Society for Clinical Nutrition and Metabolism criteria, and Global Leadership Initiative on Malnutrition criteria in patients with colorectal cancer: a multicenter study utilizing Bayesian inference
Source: Front Nutr. 2026 Feb 16;12:1671154. doi: 10.3389/fnut.2025.1671154 (PMC12950724; doi:10.3389/fnut.2025.1671154)
Supplement: Supplementary file 3 [file Table_3.docx]

Supplementary Table 3. Patient demographics and baseline characteristics (Patients with complete data).

| **Variable** | **Overall (N = 2,881)**^1^ |
| --- | --- |
| **Age** |  |
| ˂65 | 1,205.0 (41.8%) |
| ≥65 | 1,676.0 (58.2%) |
| **Sex** |  |
| male | 1,735.0 (60.2%) |
| famale | 1,146.0 (39.8%) |
| **Diabetes** |  |
| No | 2,599.0 (90.2%) |
| Yes | 282.0 (9.8%) |
| **Hypertension** |  |
| No | 2,273.0 (78.9%) |
| Yes | 608.0 (21.1%) |
| **Coronary heart disease** |  |
| No | 2,771.0 (96.2%) |
| Yes | 110.0 (3.8%) |
| **Smoking** |  |
| No | 1,782.0 (61.9%) |
| Yes | 1,099.0 (38.1%) |
| **Alcohol** |  |
| No | 2,364.0 (82.1%) |
| Yes | 517.0 (17.9%) |
| **Tea** |  |
| No | 2,162.0 (75.0%) |
| Yes | 719.0 (25.0%) |
| **Family history of cancer** |  |
| No | 2,431.0 (84.4%) |
| Yes | 450.0 (15.6%) |
| **Metastasis** |  |
| No | 2,161.0 (75.0%) |
| Yes | 720.0 (25.0%) |
| **Total protein (g/L)** |  |
| ≥35 | 2,866.0 (99.5%) |
| ˂35 | 15.0 (0.5%) |
| **Albumin (g/L)** |  |
| ≥20 | 2,872.0 (99.7%) |
| ＜20 | 9.0 (0.3%) |
| **Creatinine (umol/L)** |  |
| ≤115 | 2,807.0 (97.4%) |
| ＞115 | 74.0 (2.6%) |
| **Total Bilirubin (umol/L)** |  |
| ≤34.2 | 2,810.0 (97.5%) |
| ˃34.2 | 71.0 (2.5%) |
| **AST (U/L)** |  |
| ≤40 | 2,575.0 (89.4%) |
| ＞40 | 306.0 (10.6%) |
| **ALT (U/L)** |  |
| ≤100 | 2,825.0 (98.1%) |
| ˃100 | 56.0 (1.9%) |
| **Hemoglobin (g/L)** |  |
| ≥110 | 2,188.0 (75.9%) |
| ˂110 | 693.0 (24.1%) |
| **White Blood Cell (10^9/L)** |  |
| ≤10 | 2,611.0 (90.6%) |
| ˃10 | 270.0 (9.4%) |
| **Red Blood Cell (10^12/L)** |  |
| ≥4.0 | 1,945.0 (67.5%) |
| ˂4.0 | 936.0 (32.5%) |
| **Platelet (10^9/L)** |  |
| ≥100 | 2,729.0 (94.7%) |
| ˂100 | 152.0 (5.3%) |
| **TNM stage** |  |
| I | 234.0 (8.1%) |
| II | 813.0 (28.2%) |
| III | 1,157.0 (40.2%) |
| IV | 677.0 (23.5%) |
| **KPS score** |  |
| Independent | 2,565.0 (89.0%) |
| Semi-Dependent | 242.0 (8.4%) |
| Dependent | 74.0 (2.6%) |
| **PG-SGA** |  |
| Well-nourished | 2,311.0 (80.2%) |
| Malnourished | 570.0 (19.8%) |
| **ESPEN** |  |
| Well-nourished | 1,347.0 (46.8%) |
| Malnourished | 1,534.0 (53.2%) |
| **GLIM** |  |
| Well-nourished | 2,316.0 (80.4%) |
| Malnourished | 565.0 (19.6%) |

^1^n(%). AST, Aspartate Aminotransferase; ALT, Alanine Aminotransferase; KPS, Karnofsky Performance Status; PG-SGA, Patient-Generated Subjective Global Assessment; ESPEN, European Society for Clinical Nutrition and Metabolism; GLIM, the Global Leadership Initiative on Malnutrition.

Supplementary Table 4. Univariate and multivariate analyses of factors influencing CRC patient survival (Cox regression, patients with complete data, only the regression results for the three nutritional assessment tools are presented).

| Characteristic | Univariable | | | Multivariable | | |
| --- | --- | --- | --- | --- | --- | --- |
|  | HR^1^ | 95% CI^1^ | p-value | HR^1^ | 95% CI^1^ | p-value |
| PG-SGA |  |  |  |  |  |  |
| Well-nourished | - | - |  | - | - |  |
| Malnourished | 1.29 | 1.10, 1.53 | 0.037 | 1.27 | 1.06, 1.52 | 0.041 |
| ESPEN |  |  |  |  |  |  |
| Well-nourished | - | - |  | - | - |  |
| Malnourished | 1.26 | 1.08, 1.46 | 0.005 | 1.16 | 0.91, 1.45 | 0.236 |
| GLIM |  |  |  |  |  |  |
| Well-nourished | - | - |  | - | - |  |
| Malnourished | 1.43 | 1.22, 1.68 | ＜0.001 | 1.32 | 1.11, 1.58 | 0.002 |

^1^HR = Hazard Ratio, CI = Confidence Interval

No. Obs. = 2,881; PG-SGA, Patient-Generated Subjective Global Assessment; ESPEN, European Society for Clinical Nutrition and Metabolism; GLIM, the Global Leadership Initiative on Malnutrition.
